# Blood pressure can be seriously elevated during botulinum toxin A detrusor injection

**DOI:** 10.1007/s00345-025-05596-3

**Published:** 2025-04-03

**Authors:** Heinrich Schulte-Baukloh, Catarina Weiss, Burkert Pieske, Thorsten Schlomm, Bernhard Ralla, Hendrik Borgmann, Dirk Höppner, Sarah Weinberger

**Affiliations:** 1 Department of Urology, Charité– University Hospital Berlin, Berlin, Germany; 2 Urologic Practice Turmstrasse, Berlin-Mitte/ Moabit, Germany; 3 Urologic Practice Kurfürstendamm, Berlin-Charlottenburg, Germany; 4 Division of Cardiology, Deparmtent of Internal Medicine, University Medicine Rostock, Rostock, Germany; 5 Department of Urology, University Hospital Brandenburg, Brandenburg, Germany

**Keywords:** Botulinum toxin A, Detrusor injection, OAB, Neurogenic bladder, Blood pressure, Cardiovascular risk

## Abstract

**Introduction:**

Botulinum toxin A detrusor injection (BoNT/A-DI) is used in patients with overactive bladder (OAB) or neurogenic bladder due to multiple sclerosis (MS) or after spinal cord injury. The procedure is generally performed under local anaesthesia. We examined the influence of BoNT/A-DI on blood pressure, the most important autonomic parameter in awake patients, as a potential risk factor for cardiovascular events.

**Material & Methods:**

Patients with OAB or spontaneous voiding with neurogenic detrusor overactivity (NDO) due to MS in whom BoNT/A-DI was planned under local anaesthesia, vital parameters (systolic, diastolic, and mean blood pressure; heart rate; and rate pressure product [RPP]) were recorded before, during, and after the procedure. Participants with and without previously known hypertension were compared, along with those with initial versus repeat injections, with a focus on the high-risk group, which comprised the 20% of patients with the highest baseline blood pressure values.

**Results:**

Seventy patients were included (mean age: 64.0, median age: 66, range: 27–86 years), and two were excluded because their initial blood pressure values were too high. Sixty patients had OAB, and eight had NDO due to MS; twenty-two patients had a history of hypertension. A total of 40 patients received the first injection, and 28 received a repeat injection. Systolic blood pressure increased significantly by an average of 9.8 mmHg. However, in the hypertensive patients, systolic blood pressure rose by an average of 19.4 mmHg; isolated peak systolic values rose by up to 232 mmHg, and peak diastolic values rose by up to 128 mmHg. Cardiac stress (measured by rate pressure product [RPP]) in these patients increased significantly (RPP = 17.6 versus 7.2 in the non-hypertensive group). In the 20% of patients with the highest resting blood pressure values, systolic blood pressure rose to an average of 187.4 mmHg (15.1 mmHg compared with resting blood pressure), and cardiac workload increased by 17. No significant differences were observed between patients who received initial and repeat injections.

**Conclusions:**

Clinicians who administer BoNT/A-DI should monitor blood pressure during the procedure and be aware of the risk of potentially significantly elevated blood pressure values during BoNT/A DI, especially in patients with a medical history of hypertension. Significantly elevated pre-interventional blood pressure values should receive an internal medicine consultation timely before the intervention to prevent cardiovascular risks.

## Introduction

Overactive bladder (OAB) is defined as urinary urgency, often combined with increased frequency and nocturia, with or without urgency urinary incontinence, in the absence of urinary tract infection or other obvious pathologies [1]. Because of its chronic nature, OAB requires sufficient long-term treatment; however, patients’ adherence to long-term medication plans is generally substandard, especially for OAB [2]. Onabotulinumtoxin A (OnabotA) detrusor injection (DI) may be used after conservative first-line therapies (e.g. anticholinergics) have failed [3]. This therapy has been approved for idiopathic OAB since 2013, and previously, it was approved by the FDA for neurogenic detrusor overactivity (NDO) in patients with multiple sclerosis (MS) and adults and children over 5 years old with subcervical spinal cord injury in 2011 [4].

However, evidence on the efficacy and safety of OAB treatment with OnabotA in everyday, real-world settings is limited. Recently, Hamid et al. produced convincing evidence of symptom improvement in 504 patients in a prospective, observational, multinational study (GRACE) [5], and their data largely reflected the convincing results of the approval studies [6]. A significant reduction in the number of urinary incontinence episodes per day was observed, along with reductions in micturition, urgency, and the number of nocturia episodes per day. This was also reflected by a reduction in the use of OAB medications and a reduction in the number of incontinence products used [5, 7]. The procedure was performed under local anaesthesia (intravesical instillation or local anaesthetic gel), in most patients (69.5%); it was performed under sedation in 18.3% of patients and under general anaesthesia in 27.1% of patients. This indicates that anaesthesia in awake patients is preferred.

However, real-world data also involves identifying and minimising the risk of side effects and potential risks in routine, daily settings. Therefore, our study focused on potential risk factors in awake patients, in whom the OnabotA-DI was performed under local anaesthesia. While performing the injection, we focused on hemodynamic parameters such as blood pressure, heart rate, and the rate pressure product [RPP] - a measure of myocardial workload and cardiac oxygen consumption [8]. Elevated RPP has emerged as a marker of increased cardiovascular risk and adverse outcomes [9].

The questions that arose in this context were as follows. How do systolic, diastolic, and mean blood pressure; heart rate; and RPP—parameters of cardiac stress—change during OnabotA injection therapy? Can a high-risk group be identified? Are the stress parameters of patients amplified with repeated injections?

## Materials and methods

Adult female patients with idiopathic OAB or neurogenic bladder due to MS who were eligible for OnabotA-DI were asked to participate in the study. The study protocol was approved by the ethics committee of the Universitätsmedizin Berlin (ethics vote number EA4/203/22, ethics committee of the Universitätsmedizin Charité Berlin, Benjamin Franklin Campus). Participants were given sufficient time for reflection before providing written consent. Examinations included urine analysis to rule out urinary tract infection or haematuria, ultrasound of the bladder to rule out pathology or relevant residual urine, and vaginal examination to rule out a cystocele/uterine prolapse. The patients either had not tolerated anticholinergic or β_3_-sympathomimetic medication to treat their urinary bladder problems or had not experienced sufficient improvement. It was also documented whether it was a first or repeat OnabotA-DI. The anamnesis also inquired about the presence of previously known and, if necessary, treated hypertension. The patients were advised to take antihypertensive and other medication (apart from anticoagulants) on the day of the injection. On the day of the injection, patients were placed in the lithotomy position in the operating room, and their resting systolic and diastolic blood pressure (BP_sys_ and BP_dia_) and resting heart rate were taken using a Boso Medicus blood pressure monitor (BOSCH + SOHN GmbH u. Co. KG, Jungingen, Germany). Mean blood pressure (BP_mean_) was calculated according to the following formula: (2x BP_dia_ + BP_sys_)/3. Patients with a resting blood pressure > 190mmHg (systolic) or 120 mmHg (diastolic) were excluded from the intervention and referred for cardiology workup. Anaesthesia was achieved by instilling 50 mL of 2% lidocaine solution into the empty bladder and leaving this solution for 20 min. Cystoscopy was then performed with a rigid 21 Char. Instrument from Wolf Endoscopes (Richard Wolf GmbH, Knittlingen, Germany). The injection needles varied and were marginally different in needle size and length (22–27 gauge, 4–5 mm needle length). The injections were standardised and performed via a monitor that the patients could view if desired. The injection was performed with 100, 150, or 200 U of onabotulinumtoxin A (Botox^®^, AbbVie), dissolved in 10 mL NaCl (regardless of the dosage). Blood pressure and heart rate were measured repeatedly during the procedure, and the average value was calculated. The blood pressure and heart rate assessment was performed again 5 min after the procedure.

The blood pressure values (BP_sys_/ BP_dia_ in mmHg), heart rate (beats per minute, bpm), and RPP (BP_sys_ x heart rate/100) were analysed for the entire cohort, and the results were also stratified as follows: hypertensive vs. non-hypertensive, first vs. repeat injections, and the values of the 20% patients with the highest measured resting blood pressure values (high-risk group) and their proportion in the group of patients with known hypertension.

### Statistics

The following statistical analyses were performed. For metrically scaled variables, mean, standard deviation, median, and minimum and maximum values were calculated. Absolute and relative frequencies were calculated for nominal/ordinal scaled variables. To test for differences between groups, Fisher’s exact tests or Chi-square tests were used for ordinally scaled variables, and Welch’s t-tests or analysis of variance ANOVA (or their non-parametric analogues) were used for metrically scaled characteristics after checking for normality. All analyses were performed with R Core Team (2023); R: A Language and Environment for Statistical Computing; R Foundation for Statistical Computing, Vienna, Austria; R version 4.3.1 (2023-06-16 ucrt); RStudio Team (2020); RStudio: Integrated Development for R; RStudio, PBC, Boston, MA.

## Results

Seventy female patients were included (average age: 64.0 ± 15.9, median age: 66, range: 27–86 years). Two patients were excluded because their resting systolic blood pressure values were > 190 mmHg. Most had idiopathic OAB (*n* = 60; 88.2%), and eight patients had neurogenic bladder due to MS with an expanded disability status scale (EDSS) of < 6 and no need to self-catheterise the bladder. A total of 40 patients (58.8%) received the first injection, and 28 (41.2%) received a repeat injection; 59 (86.8%) patients received 100 U, 4 (5.9%) patients received 150 U, and 5 (7.4%) patients received 200 U OnabotA.

**Fig. 1:**
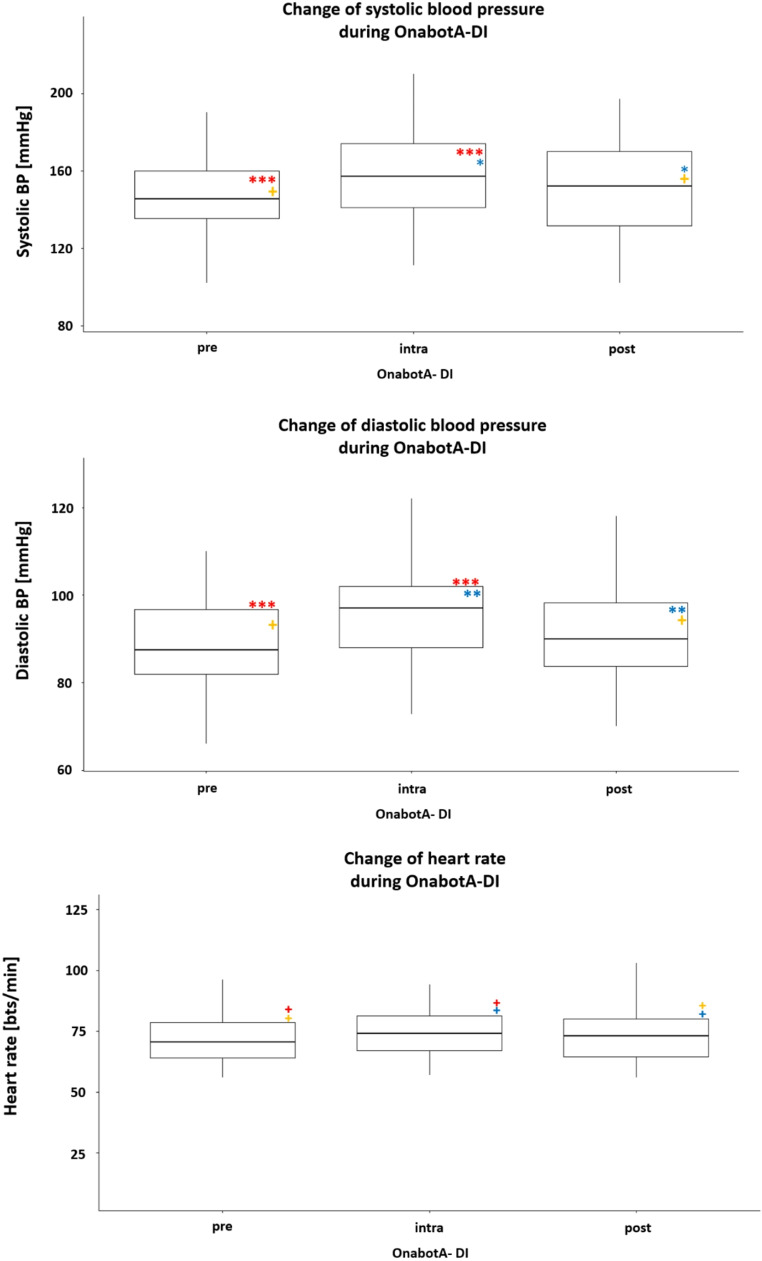
The medians of the systolic (**a**) and diastolic (**b**) blood pressure values ​​as well as the heart rate (**c**) are shown. Comparative significances between pre-intra, intra-post, pre-post injection: + not significant; one* = *p* < 0.05, two* = *p* < 0.01, three* = *p* < 0.001. OnabotA-DI: onabotulinumtoxinA detrusor injection The boxes include the 2nd and 3rd quartiles of blood pressure values ​​(thus the middle 50%). The middle lines represent the median, the vertical lines symbolize the range

*Results for the overall cohort*: Both systolic and diastolic blood pressure increased significantly by an average of 9.8 mmHg and 7.0 mmHg, respectively, compared with the preoperative and intraoperative values. Heart rate increased by an average of 1.5 bpm, but the average value did not differ significantly between measurement times (Fig. 1a-c).

*Results between patients with and without previously known hypertension*: Blood pressure values changed much more dramatically in patients with previously known hypertension. In these patients, systolic blood pressure increased significantly by 19.4 mmHg compared with the pre- and intraoperative values, with occasional systolic peak values of up to 232 mmHg and diastolic values of up to 128 mmHg. The same was true for mean blood pressure, which is shown in Table 1 (instead of diastolic BP). The RPP also reflected significant additional strain on the heart, particularly in patients with hypertension who had cardiac pre-stress; in these patients, RPP increased by 17.6 compared with 7.2 in patients without hypertension.

**Table 1:**
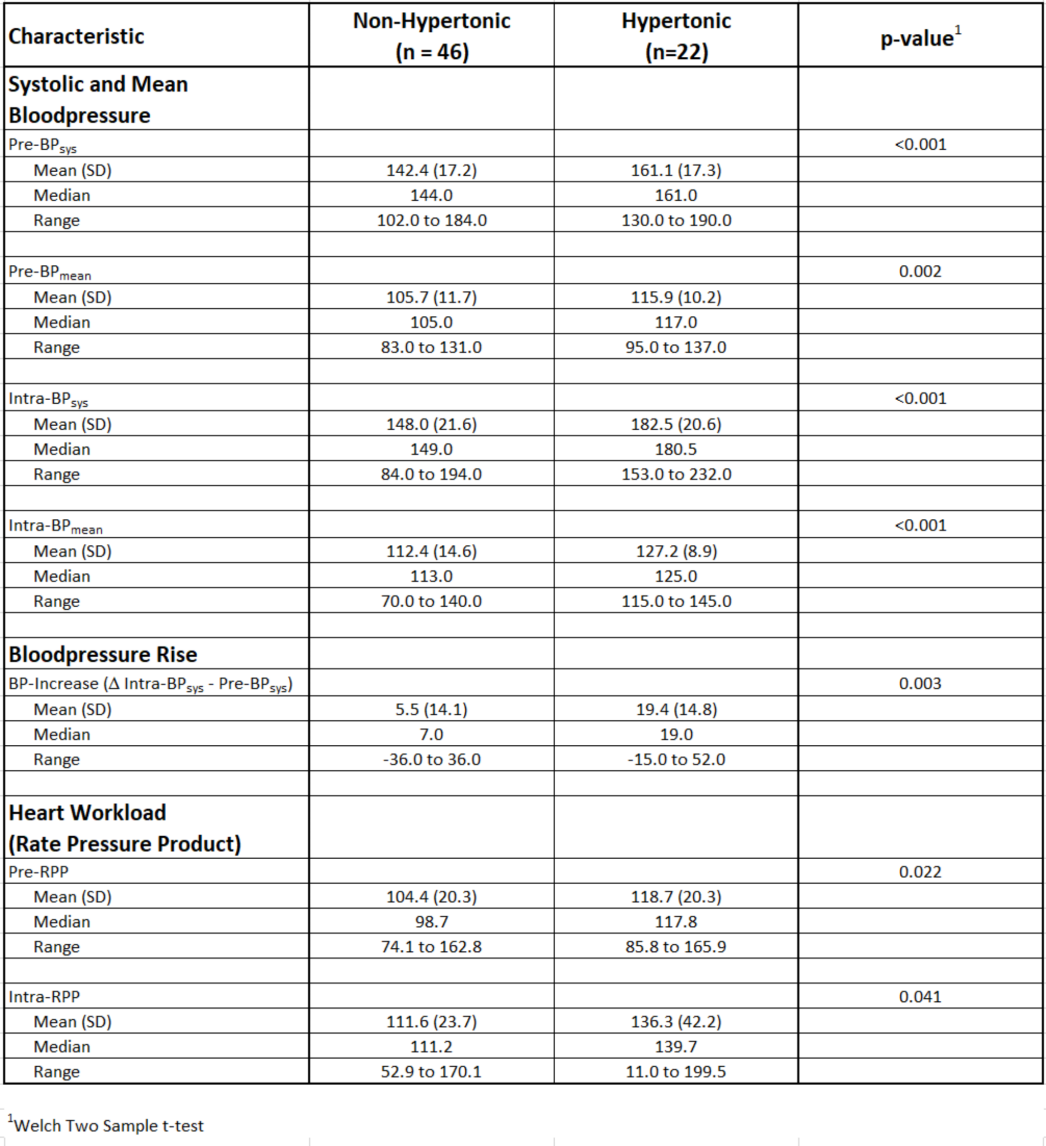
Blood pressure results of patients with and without previously known hypertension. Pre-/ Intra-BP_sys_: systolic blood pressure before/ during intervention; Pre-/ Intra-BP_mean_: mean blood pressure before/ during intervention; RPP: rate pressure product

*Results of the 20% of patients with the highest resting blood pressure values (high-risk group)*: Of the 20% of patients with the highest preoperative blood pressure values at rest (*n* = 14 patients), five did not have previously known hypertension and nine had previously known hypertension. In these 14 patients, the comparison of the pre- and intraoperative systolic blood pressure values showed an increase of 15.5 mmHg (from 172.9 ± 9.3 to 187.4 ± 19.6; *p* = 0.00607) and an increase in cardiac strain/heart workload (RPP) of 17 (from 118.3 ± 15.1 to 135.3 ± 25.2; *p* = 0.00392). Furthermore, the 10% of patients with the highest resting blood pressure values (*n* = 7, of whom 2 were in the non-hypertensive group and 5 were from the hypertensive group) showed average intraoperative blood pressure values of 196.0 ± 22.5 (median: 194.0, range: 162.0–232.0) mmHg.

### Results between initial and repeat injections

When patients were stratified according to first or repeat injections, no significant differences were observed in pre- and intraoperative BP_sys−_ or cardiac stress (RPP) in hypertensive or non-hypertensive patients.

### Side effects

No patients experienced adverse cardiovascular events during or after the procedure.

## Discussion

OnabotA-DI has been used for years in a standardised outpatient setting under local anaesthesia for patients with idiopathic OAB or neurogenic bladder due to MS and other underlying neurogenic diseases [4]. Although the procedure is tolerated very well by most patients, some patients fear the procedure, especially the pain that may associated with it; thus, this has been the subject of several studies [10].

To our knowledge, no studies have systematically reported changes in hemodynamic parameters including blood pressure, heart rate, and RPP, during the OnabotA- DI procedure. Alongside systolic and mean BP, cardiac strain (RPP) reflects the stress and risk for the heart and cardiovascular events [9]. Here, an absolute increase of > 10 (in our study the difference between the non- / hypertensive groups, RPP increased by 17.6 in the hypertensive compared with 7.2 in patients without hypertension) indicates a clinically meaningful rise in cardiac strain [9].

Hypertension, stress, and cardiac strain are a sum of different components:

### White coat hypertension

White coat hypertension is an increase in blood pressure that is almost always observed and is caused by patients’ excitement and fear of doctors and medical procedures [11]. Thus, the resting blood pressure we measured before the procedure corresponds more to elevated blood pressure observed during stress or exercise (i.e. white coat hypertension) than to actual resting blood pressure (as named in our study). On the other hand, resting blood pressure measured under home conditions, for example, does not necessarily reflect the predominant blood pressure of a patient but rather their blood pressure under *unusually* calm conditions, which could underestimate the presence of substantial hypertension; this is known as masked hypertension [12]. Interestingly, in our study, stratifying the blood pressure values and cardiac strain (RPP) according to the first versus repeat injections did not reveal any differences between our patient groups, although this was expected at least partially in the case of repeat injections, during which the patients know the doctor and know what to expect.

Obviously, white coat hypertension can only be marginally influenced.

### Hypertension due to anxiety about the procedure

We have previously shown that the anxiety from the OnabotA-DI under local anaesthesia is understandably present [13]. This anxiety may be reduced if the patients have a more detailed understanding (e.g. through video demonstration) of what they can expect during the procedure. Karalar et al. [14] recently accomplished this, showing that patients reported lower levels of fear and anxiety when shown a video of a ureteroscopy before the procedure rather than simply having the procedure described to them verbally.

Thus, hypertension due to anxiety might be partially ameliorated by providing patients with better, visually based pre-operative information.

#### Hypertension due to pain

The level of pain, which does not fundamentally change between the first and second injection, appears to have a significant influence on the change in blood pressure. Thus, reducing the pain would be a beneficial approach. In a review, Faure Walker et al. [10] concluded that reducing the injection sites, alkalinising the lidocaine anaesthesia (which increases the permeability of the urinary bladder mucosa), and carrying out electromotive drug administration (EMDA) therapy helped patients accept the procedure in terms of pain [10, 15, 16].

Apparently, pain reduction is associated with reduced blood pressure.

#### Hypertension due to bladder filling

However, an increase in blood pressure can also be caused solely by a fuller urinary bladder; this occurs not only in patients with paraplegia, in whom the phenomenon of autonomic dysreflexia [17] is well-known (this did not occur in our patient population because of the inclusion criteria) but also in patients without neurogenic bladder. For example, Fagius et al. [18] demonstrated that systolic and diastolic blood pressure increased significantly as the bladder filled (by 15 mmHg and 10 mmHg, respectively) and both blood pressure parameters returned to the initial values after micturition. Functionally, a vesicovascular response mediated by sympathetic vasoconstrictor neurons was discussed. Choi et al. [19] reported similar significant results, but with lower increases in systolic and diastolic blood pressure (4.2 and 2.8 mmHg), regarding bladder filling in middle-aged women. Furthermore, considerable increases in blood pressure with an increasingly full urinary bladder were found during hydrodistension of the urinary bladder in patients with bladder pain syndrome [20]. Blood pressure and heart rate during hydrodistension were monitored in patients with bladder pain syndrome who had typical interstitial cystitis endoscopic findings, and interestingly, this was assessed under general anaesthesia; the researchers found that the systolic and diastolic blood pressure increased by 25 ± 19 and 21 ± 12 mm Hg, respectively, and average heart rate increased by 12 ± 11 beats per minute.

Thus, the influence of bladder filling on blood pressure might be partially ameliorated by reduced bladder filling (e.g. half or two-thirds of the bladder capacity).

In our study, some patients were found to have initially and sometimes significantly elevated blood pressure (the 20% of patients with the highest blood pressure values; i.e. the high-risk group), which urologists typically overlook in the routine procedure because they do not record this parameter in a standardised manner during OnabotA-DI. Notably, however, this initially elevated blood pressure continued to increase statistically and clinically significantly to average values of 187.4 mmHg during the procedure. Importantly, increases in blood pressure, even if they are very short-term, carry an increased CVD and mortality risk [21].

According to the European guidelines, the following blood pressure categories apply (Table 2, Appendix [22]). In the American Heart Association guidelines, even the upper blood pressure limits are worded more restrictively (Table 3, Appendix [23, 24]). In the latter, the following blood pressure values are formulated with regard to white coat hypertension: in adults with an untreated BP_sys_ greater than 130 mmHg but less than 160 mmHg or a BP_dia_ greater than 80 mm Hg but less than 100 mm Hg, it is reasonable to screen for the presence of white coat hypertension [23].

This means that all of our patients in the high-risk group (the 20% of patients with the highest measured resting blood pressure values) started the procedure with significantly elevated blood pressure according to European and American guidelines. This was true not only for patients with previously known hypertension (*n* = 9) but also without previously known hypertension (*n* = 5). Additionally, blood pressure increased intraoperatively in almost all patients to category 2 or 3 (according to the European guidelines [22]); the average value in this group was 187.4 mmHg.

The main findings of our study can be summarised as follows:

- Unsurprisingly, blood pressure increased significantly during the OnabotA-DI. However, as urologists, we must be aware of these increases in blood pressure, which can sometimes be serious.
- Caution is advised in patients with known hypertension, along with patients with significantly elevated resting blood pressure before the procedure (therefore, it is crucial to measure it). The stress caused by this procedure, although only short-term, corresponds to the increase in blood pressure under very strenuous exertion, and an increased risk of cerebrovascular accident (CVA) occurrence, even under short-term blood pressure crises, has been reported elsewhere.
- In practice, patients should take their antihypertensive drugs on the day of the injection procedure. Ideally, patients with a resting pressure of 160mmHg (blood pressure category 2 according to the European guidelines) should be presented to an internist or cardiologist before OnabotA-DI is performed if the urologist does not feel confident starting the patient on antihypertensive therapy.

## Conclusions

Many medical interventions require the monitoring of vital signs such as blood pressure and heart rate; however, during OnabotA-DI, this practice is critical for patients with or without a previous diagnosis of hypertension, especially if patients have previous cardiovascular damage (e.g. with heart failure, coronary heart or valve disease). There could be an increased risk during the procedure and it is good to be prepared for this. Monitoring these stress parameters before the procedure not only allows clinicians to detect the presence of “white coat” or anxiety-related hypertension but also enables them to evaluate the cardiac stress of the patient and the necessity of internal medical consultation and anti-hypertensive medication.

## Data Availability

No datasets were generated or analysed during the current study.

## Appendix

### Classification of blood pressure and definitions of hypertension grade

**Table 2:** ESC/ESH GUIDELINES [22]

Category	Systolic (mmHg)		Diastolic (mmHg)
Optimal	< 120	and	< 80
Normal	120–129	and/or	80–84
High normal	130–139	and/or	85–89
Grade 1 hypertension	140–159	and/or	90–99
Grade 2 hypertension	160–179	and/or	100–109
Grade 3 hypertension	> 180	and/or	> 110

**Table 3:** ACC/AHA guidelines [23]

BP Category	SBP		DBP
Normal	< 120mmHg	and	< 80mmHg
Elevated	120–129 mmHg	and	< 80mmHg
**Hypertension**			
Stage I	130–139 mmHg	or	80-89mmHg
Stage II	≥ 140mmHg	or	≥ 90mmHg
